# The mediating and moderating roles of achievement goal orientation in the relationship between AI writing feedback and English writing self-efficacy among Chinese university students

**DOI:** 10.3389/fpsyg.2026.1860723

**Published:** 2026-07-31

**Authors:** Xiangyu Kan, Lin Li

**Affiliations:** 1 Department of Basic Courses, Tianjin Vocational University, Tianjin, China; 2 School of Foreign Languages, Xinxiang University, Xinxiang, Henan, China

**Keywords:** achievement goal orientation, automated writing feedback, moderated mediation, second language writing, writing self-efficacy

## Abstract

This study investigates how AI-generated writing feedback influences English writing self-efficacy among Chinese university students and examines the dual role of achievement goal orientation as both a mediator and a moderator in this relationship. A quasi-experimental pretest-posttest design was implemented with 148 s-year English majors over a 12-week semester. Participants in the experimental group received feedback from a GPT-4-based automated evaluation platform, while the control group received conventional teacher feedback. Writing self-efficacy, achievement goal orientation, and AI feedback perception were measured at both time points. Results indicated that AI feedback significantly enhanced writing self-efficacy (*d* = 0.94), with the largest gains in ideation and self-regulation. Mediation analysis revealed that mastery-approach goal orientation partially mediated the feedback–efficacy relationship, accounting for 33.4% of the total effect, whereas performance-approach orientation did not function as a significant mediator. Moderation analysis showed that mastery-approach orientation amplified the positive effect of AI feedback on self-efficacy, while performance-approach orientation attenuated it. A moderated mediation model confirmed that the indirect effect through mastery-approach goals varied across learner motivational profiles. These findings extend social cognitive theory into AI-mediated pedagogical contexts and suggest that instructors should incorporate goal-framing activities to maximize the efficacy-building potential of AI writing tools.

## 1 Introduction

The integration of artificial intelligence into second-language writing instruction has reshaped how feedback is delivered, processed, and acted upon in university settings. Automated writing evaluation systems—ranging from rule-based grammar checkers to large language model-powered tutors—now offer students immediate, individualized commentary on lexical accuracy, syntactic complexity, coherence, and argumentation (Shi and Aryadoust, 2024). This shift from exclusively human-mediated feedback to AI-assisted response has prompted educators and researchers alike to ask not merely whether such tools improve writing quality, but how they alter the psychological dispositions that sustain long-term writing development (Song and Song, 2023).

Among these dispositions, writing self-efficacy—a learner’s confidence in their capacity to plan, draft, and revise texts at a given standard—stands out as a robust predictor of writing persistence, strategy use, and ultimate achievement (Pajares, 2003). Bandura’s (1997) social cognitive theory positions self-efficacy as shaped by four principal sources: mastery experience, vicarious experience, social persuasion, and physiological-affective states. Feedback, particularly evaluative feedback from a perceived authority, operates primarily through social persuasion and, when it enables successful revision, through mastery experience as well (Schunk and DiBenedetto, 2020). Yet most empirical work on feedback and self-efficacy has examined human tutors or peer reviewers; whether AI-generated feedback activates the same efficacy-building pathways remains an open and theoretically interesting question (Escalante et al., 2023).

A handful of quasi-experimental studies have compared AI feedback with teacher feedback on writing performance metrics such as grammatical accuracy and organizational coherence (Wei et al., 2023). Others have surveyed student perceptions of automated feedback credibility and usefulness (Ding and Zou, 2024). What these lines of inquiry share is a predominant focus on outcome variables—scores, error counts, satisfaction ratings—at the expense of process-level psychological mechanisms. We know relatively little, in other words, about the cognitive-motivational routes through which AI feedback comes to strengthen or weaken a student’s belief in their own writing competence. This gap matters because self-efficacy is not a fixed trait; it is continually negotiated through the learner’s interpretation of performance information, and that interpretation is filtered through pre-existing motivational orientations (Zumbrunn et al., 2020).

Achievement goal theory offers a compelling lens for understanding such filtering. The framework distinguishes between mastery-approach goals, where learners seek to develop competence, and performance-approach goals, where the aim is to demonstrate competence relative to others (Elliot and McGregor, 2001). A student oriented toward mastery is more likely to treat corrective feedback—whether from a human or an algorithm—as diagnostic information that guides improvement, thereby reinforcing self-efficacy. A performance-oriented student, by contrast, may perceive the same feedback as an external judgment of ability, triggering defensive processing and potentially eroding confidence (Keller et al., 2024). Achievement goal orientation could therefore function both as a mediator, translating the informational content of AI feedback into efficacy beliefs through distinct appraisal pathways, and as a moderator, conditioning the strength or direction of the feedback–efficacy link depending on which goal profile predominates (Liu et al., 2023).

Despite the theoretical plausibility of this dual role, existing studies have rarely examined achievement goals in the context of automated writing feedback. Research on goal orientation and feedback processing has been conducted mainly in face-to-face classroom experiments with human evaluators (Ma et al., 2022), and the few investigations that do involve technology-mediated feedback tend to treat goal orientation as a simple covariate rather than modeling its mediating and moderating functions simultaneously (Zhang and Hyland, 2018). This leaves a notable theoretical vacancy: we lack an integrated account of how AI writing feedback influences self-efficacy through, and contingent upon, learners’ goal orientations.

The present study addresses this vacancy with three objectives. First, we estimate the direct effect of AI writing feedback on English writing self-efficacy among Chinese university students enrolled in an academic English course. Second, we test whether mastery-approach and performance-approach goal orientations mediate the relationship between AI feedback and self-efficacy, hypothesizing that AI feedback promotes mastery goals, which in turn enhance efficacy beliefs. Third, we examine whether the same goal orientations moderate the feedback–efficacy path, such that the benefit of AI feedback is amplified for mastery-oriented learners and attenuated for performance-oriented ones. A moderated mediation model is constructed and tested using structural equation modeling with bootstrapped confidence intervals (Hayes, 2022).

By embedding achievement goal orientation within the AI feedback–self-efficacy nexus, this research makes three contributions. Theoretically, it extends Bandura’s efficacy framework into the domain of human–AI pedagogical interaction, specifying the motivational channels through which algorithmic feedback acquires psychological meaning. Methodologically, it moves beyond the prevalent pre-post comparison design by modeling mediation and moderation within a single analytical framework. Practically, the findings can inform how writing instructors calibrate AI feedback delivery—for instance, by coupling automated commentary with goal-framing prompts—to maximize its efficacy-building potential across diverse learner profiles.

## 2 Theoretical foundations and research hypotheses

### 2.1 Core concepts and theoretical framework

AI writing feedback, as conceptualized here, refers to computer-generated evaluative and corrective commentary on student texts, produced by natural language processing algorithms that assess surface-level accuracy, discourse organization, and rhetorical effectiveness (Zhai and Ma, 2022). Such feedback may be directive (supplying corrections), informational (diagnosing error patterns), or scaffolding-oriented (posing revision-prompting questions), and these typological distinctions matter because each type activates different learner processing routines (Hattie and Timperley, 2007).

Self-efficacy theory, rooted in Bandura’s (1997) social cognitive framework, holds that individuals’ beliefs about their own capabilities determine the tasks they attempt, the effort they invest, and their resilience when encountering difficulty. In second-language writing, efficacy beliefs have been operationalized across sub-domains—ideation, convention control, self-regulation—and each sub-domain responds differently to external evaluative input (Bruning et al., 2013). Feedback that makes successful revision tangible can generate mastery experience, the most potent efficacy source, whereas feedback perceived as judgmental without actionable guidance may heighten anxiety and dampen confidence (Ekholm et al., 2015).

The motivational filter through which learners interpret such feedback can be mapped onto Elliot and McGregor’s 2 × 2 achievement goal model, which crosses the mastery–performance distinction with approach–avoidance valence (Elliot and McGregor, 2001). Mastery-approach goals orient learners toward deepening competence; mastery-avoidance goals drive them to avoid misunderstanding or skill loss. Performance-approach goals center on outperforming peers, while performance-avoidance goals aim at escaping unfavorable social comparison (Elliot, 2005). Each orientation predisposes learners to construe identical feedback differently: mastery-approach students tend to engage analytically with corrective input, performance-avoidance students tend to dismiss or withdraw from it (Dweck and Leggett, 1988).

The theoretical linkage we propose is straightforward but, to our knowledge, untested in the AI feedback domain. AI feedback constitutes an informational stimulus whose impact on writing self-efficacy is not direct but channeled through the goal orientation that shapes how learners appraise, internalize, or reject that information (Lipnevich and Smith, 2009). Goal orientation thus occupies a dual structural position—transmitting feedback effects on efficacy (mediation) and altering the magnitude of those effects across learner profiles (moderation).

### 2.2 Theoretical derivation of mediation and moderation effects

Social cognitive theory predicts that external feedback reshapes self-efficacy not in a vacuum but through cognitive appraisal processes that are themselves shaped by motivational dispositions (Bandura, 1986). Control-value theory adds a complementary claim: the subjective value a learner assigns to an evaluative event determines whether that event triggers approach or avoidance behavior (Pekrun, 2006). Together, these frameworks suggest a two-step causal sequence in which AI feedback first activates or strengthens a particular goal orientation, which then channels the feedback signal into efficacy beliefs. The mediation path can be formalized following Baron and Kenny’s (1986) conditions: the independent variable (AI feedback) must predict the mediator (goal orientation), the mediator must predict the dependent variable (self-efficacy) controlling for the independent variable, and the direct path must attenuate when the mediator enters. In standard regression notation, these relations are specified in Equations 1, 2:

$$M=\alpha_{0}+\mathrm{aX}+\varepsilon_{1}$$ (1)

$$Y=\beta_{0}+c^{\prime }X+\mathrm{bM}+\varepsilon_{2}$$ (2)

where X denotes AI feedback intensity, M represents goal orientation, Y is writing self-efficacy, a and b capture the indirect path, and $c^{\prime }$ is the residual direct effect (Baron and Kenny, 1986).

The moderation logic rests on a different premise—that goal orientation does not merely transmit the feedback effect but amplifies or dampens it depending on its type. Mastery-approach learners, who interpret corrective input as a growth signal, should exhibit a stronger feedback–efficacy link than performance-avoidance learners, for whom the same input may feel threatening (Pintrich, 2000). The conditional direct effect is expressed as shown in Equation 3:

$$Y=\gamma_{0}+c_{1}X+c_{2}W+c_{3}(X\times W)+\varepsilon_{3}$$ (3)

here W is the moderator (goal orientation) and $c_{3}$ captures the interaction (Aiken and West, 1991). When mediation and moderation operate simultaneously, a moderated mediation model is appropriate, with the conditional indirect effect given by Equation 4:

$$\theta (W)=a\times (b_{1}+b_{2}W)$$ (4)

This expression, derived from Hayes’s (2022) integrative framework, allows the indirect effect to vary across levels of the moderator. We adopt this formulation to test whether the mediating role of goal orientation itself fluctuates with the learner’s predominant goal profile—a question that neither pure mediation nor pure moderation models can address alone.

### 2.3 Research hypotheses and conceptual model

Drawing on the theoretical architecture established above, we advance four hypotheses. The first concerns the direct path: AI writing feedback, by supplying actionable diagnostic information and enabling iterative revision, should elevate students’ writing self-efficacy (H1) (Schunk and DiBenedetto, 2020). The full structural model expresses this direct effect alongside the mediated and moderated paths, as presented in Equation 5:

$$\begin{array}{l} Y=\delta_{0}+c^{\prime }X+b_{1}M_{\mathrm{map}}+b_{2}M_{\mathrm{pap}}+b_{3}(X\times M_{\mathrm{map}})\\ +b_{4}(X\times M_{\mathrm{pap}})+\varepsilon_{4} \end{array}$$ (5)

Where $M_{\mathrm{map}}$ and $M_{\mathrm{pap}}$ represent mastery-approach and performance-approach orientations, respectively, and $b_{3}$ and $b_{4}$ are the interaction coefficients capturing moderation (Hayes, 2022).

For the mediation hypotheses, we expect AI feedback to strengthen mastery-approach orientation, which in turn bolsters self-efficacy (H2a), while its effect on performance-approach orientation—and the downstream consequence for efficacy—should be weaker or nonsignificant (H2b) (Pekrun et al., 2009). The indirect effects through each mediator are estimated as shown in Equation 6:

$$IE_{\mathrm{map}}=a_{1}\times b_{1},IE_{\mathrm{pap}}=a_{2}\times b_{2}$$ (6)

where $a_{1}$ and $a_{2}$ are the regression weights from feedback to each goal orientation (Baron and Kenny, 1986).

The moderation hypotheses posit that mastery-approach orientation amplifies the positive feedback–efficacy relationship (H3a: $b_{3}>0$ ), whereas performance-avoidance orientation attenuates it (H3b: the analogous interaction term for avoidance orientation is negative) (Elliot and Church, 1997). The conditional indirect effect, integrating both mechanisms, takes the form given in Equation 7:

$$\theta_{j}(W)=a_{j}\times (b_{j}+b_{j+2}W),j=1,2$$ (7)

This expression permits us to probe the indirect effect at different levels of each moderator, following the index of moderated mediation recommended by Hayes (2022). The resulting conceptual model thus specifies three testable layers—direct effect, parallel mediation through two goal orientations, and cross-level moderation of both the direct and indirect paths—within a single integrated framework (Preacher et al., 2007).

## 3 Research design and methods

### 3.1 Experimental design and participant selection

**Figure 1:**
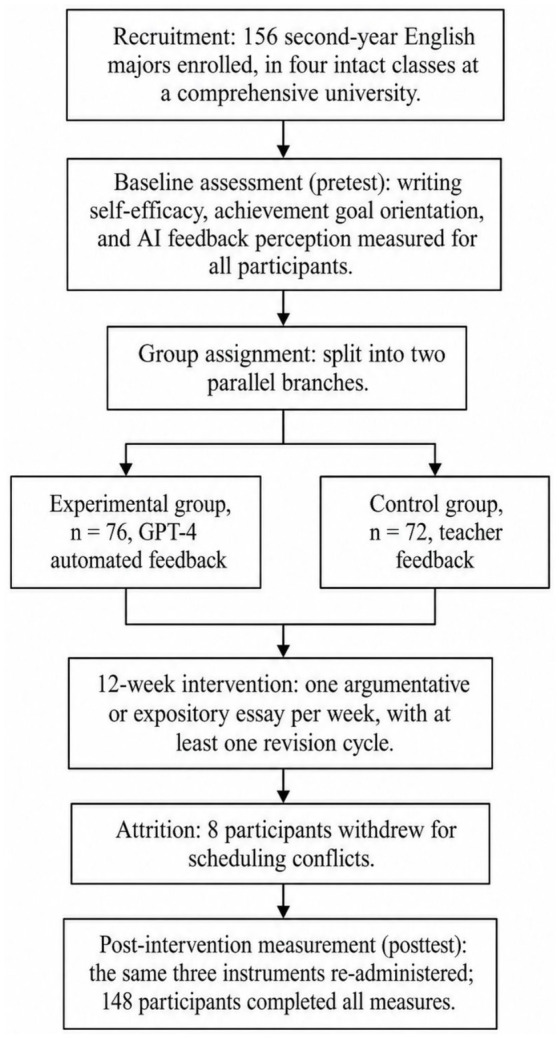
Experimental procedure spanning recruitment, baseline assessment, 12-week intervention, and post-intervention data collection, with participant attrition noted at each stage.

A quasi-experimental pretest-posttest nonequivalent control group design was adopted. Four intact classes of second-year English majors at a comprehensive university in eastern China were assigned—two to the experimental condition receiving AI-generated writing feedback via a GPT-4-based automated evaluation platform, and two to the control condition receiving conventional teacher-only feedback. Both groups completed identical argumentative and expository writing tasks across a 12-week semester, with one essay assignment per week. Figure 1 illustrates the procedural flow from recruitment through post-intervention measurement.

To make the procedure reproducible, a few operational details deserve spelling out. The automated platform was built on GPT-4 and reached through a browser-based interface hosted on the university intranet; after submitting a draft, each student in the experimental group received, usually within about a minute, structured commentary organized under four headings—content and argument, organization and coherence, language accuracy, and a short set of revision-prompting questions—and was asked to revise and resubmit at least once before the weekly deadline. The eight writing prompts, two per month, were taken from the course syllabus and covered familiar argumentative and expository topics, such as the value of a gap year, the trade-offs of remote work, and whether universities should keep weighting standardized tests. Drafting and revision were done online and outside class hours, so that the two conditions worked under identical time and topic constraints; the weekly 90-minute class meetings were kept for instruction shared by all four classes.

Control-group essays were marked by the same two instructors with a shared analytic rubric—content and argument (30 points), organization and coherence (30), language accuracy (25), and mechanics (15), summing to 100—together with margin comments and a brief end note. The automated platform was calibrated against this same rubric, so the two feedback streams differed in their source rather than in the scoring frame they applied. Inter-rater agreement on a 20-essay subset was strong (ICC = 0.87). The three questionnaires were delivered online through a secure survey link during the first and the final class sessions of the semester, took roughly 15 min to finish, and were completed anonymously under a code-matched ID so that pretest and posttest responses could be paired without identifying any individual.

Sample size was determined *a priori* using G*Power 3.1 (Faul et al., 2009). Assuming a medium effect size ( $f^{2}=0.15$ ), α=0.05 , and desired power of 0.80 for a regression model with six predictors, the minimum required sample was obtained from Equation 8:

$$N\geq \frac{L}{\lambda }+k+1=\frac{12.83}{0.15}+6+1\approx 93$$ (8)

where L is the tabled critical value for the specified power and k is the number of predictors (standard G*Power formula) (Cohen, 1992). To buffer against attrition, we enrolled 156 students; 148 completed all measures after eight withdrew for scheduling conflicts.

Table 1 summarizes the demographic profile of the final sample. The gender imbalance toward female participants reflects the broader enrollment pattern in English-major programs at Chinese universities (Gu and Benson, 2023).

**Table 1:** Demographic characteristics of participants by group.

Characteristic	Experimental (*n* = 76)	Control (*n* = 72)	Total (*N* = 148)
Female, *n* (%)	58 (76.3)	53 (73.6)	111 (75.0)
Male, *n* (%)	18 (23.7)	19 (26.4)	37 (25.0)
Mean age (SD)	19.8 (0.91)	20.1 (0.87)	19.9 (0.89)
CET-4 mean score (SD)	512.4 (38.6)	508.7 (41.2)	510.6 (39.8)
Prior AI tool use, *n* (%)	31 (40.8)	28 (38.9)	59 (39.9)
First-generation college, *n* (%)	22 (28.9)	25 (34.7)	47 (31.8)

Baseline CET-4 scores and prior AI tool exposure did not differ between groups (independent-samples *t*-test and Chi-square test, all *p* > 0.05), confirming pre-intervention equivalence. The study received approval from the university’s Institutional Review Board, and all participants provided written informed consent before enrollment (American Psychological Association, 2002).

### 3.2 Instruments and variable measurement

Three self-report instruments were administered at both pre- and post-intervention time points. Writing self-efficacy was measured with a 16-item scale adapted from Bruning et al.’s (2013) Writing Self-Efficacy Index, covering three sub-dimensions: ideation (6 items), convention control (5 items), and self-regulation (5 items). Items were rated on a 7-point Likert scale ranging from 1 (*no confidence at all*) to 7 (*complete confidence*), and the Chinese translation underwent back-translation verification by two bilingual applied linguists. To give a sense of the wording, a representative ideation item reads, “I can think of enough ideas to develop my essay fully,” and a self-regulation item asks how sure students are that they “can keep revising until the writing meets the standard I have set.”

Achievement goal orientation was assessed with Elliot and McGregor’s 12-item Achievement Goal Questionnaire-Revised, which yields four 3-item subscales corresponding to the 2 × 2 framework: mastery-approach, mastery-avoidance, performance-approach, and performance-avoidance (Elliot and McGregor, 2001). Each item was scored on a 5-point scale. We developed the AI Writing Feedback Perception Scale (AIWFPS) specifically for this study, drawing item stems from prior work on automated feedback acceptance (Stevenson and Phakiti, 2014). The 10-item instrument comprises two dimensions—perceived informativeness (6 items) and perceived credibility (4 items)—scored on a 5-point agreement scale. By way of illustration, a mastery-approach item reads, “My goal is to learn as much as I can in this writing course,” whereas a performance-approach item reads, “I want to write better than the other students in my class.” On the AIWFPS, a sample informativeness item is “The feedback made clear what I needed to improve in my draft,” and a sample credibility item is “I trust the judgments the system made about my writing.”

A pilot study with 62 students from a non-participating cohort established preliminary psychometric properties. Internal consistency was evaluated via Cronbach’s α , and construct validity was assessed through confirmatory factor analysis (CFA). Model fit was judged against standard thresholds (Hu and Bentler, 1999): comparative fit index (CFI) ≥ 0.90, root mean square error of approximation (RMSEA) ≤ 0.08, and standardized root mean square residual (SRMR) ≤ 0.08. Composite reliability (CR) and average variance extracted (AVE) were computed using the standard formulas given in Equations 9, 10:

$$\mathrm{CR}=\frac{{\left(\sum_{i=1}^{p}\lambda_{i}\right)}^{2}}{{\left(\sum_{i=1}^{p}\lambda_{i}\right)}^{2}+\sum_{i=1}^{p}\delta_{i}}$$ (9)

$$\mathrm{AVE}=\frac{\sum_{i=1}^{p}\lambda_{i}^{2}}{p}$$ (10)

where $\lambda_{i}$ is the standardized factor loading for item i , $\delta_{i}$ is the error variance, and p is the number of indicators per factor (Fornell and Larcker, 1981). As presented in Table 2, all subscales exceeded the 0.70 threshold for CR, and AVE values surpassed 0.50, confirming adequate convergent validity (Hu and Bentler, 1999).

**Table 2:** Reliability and validity indices for all measurement scales (pilot study, *n* = 62).

Scale/Subscale	Items	Cronbach’s α	CR	AVE	CFI	RMSEA
Writing SE: ideation	6	0.89	0.90	0.60	0.96	0.062
Writing SE: convention	5	0.85	0.86	0.55	0.95	0.058
Writing SE: self-regulation	5	0.87	0.88	0.59	0.97	0.051
AGO: mastery-approach	3	0.82	0.83	0.62	0.99	0.039
AGO: performance-approach	3	0.80	0.81	0.58	0.98	0.044
AIWFPS: informativeness	6	0.86	0.87	0.53	0.94	0.067
AIWFPS: credibility	4	0.83	0.84	0.57	0.96	0.055

**Figure 2:**
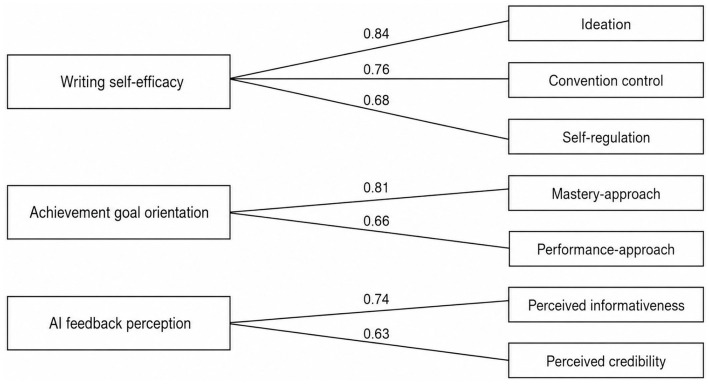
Confirmatory factor analysis measurement model showing three latent constructs (writing self-efficacy, achievement goal orientation, AI feedback perception) with standardized factor loadings for each observed indicator.

Figure 2 depicts the measurement model with latent factors and their observed indicators. All standardized factor loadings ranged from 0.63 to 0.88, and no cross-loadings exceeded 0.30, supporting discriminant validity across the seven subscales (Hair et al., 2019).

### 3.3 Data analysis strategy

Data analysis proceeded in four stages. Descriptive statistics and independent-samples *t*-tests first established group equivalence at baseline and quantified pre-post change. Pearson correlations among all study variables were then computed to verify the bivariate associations presupposed by the structural model.

Mediation was tested via the PROCESS macro (Model 4) with 5,000 bootstrap resamples (Hayes, 2022). The indirect effect and its 95% bias-corrected confidence interval were estimated as shown in Equations 11, 12:

$$\mathrm{ab}=\hat{a}\times \hat{b}$$ (11)

$${\mathrm{CI}}_{95\%}=[ab_{\alpha /2},ab_{(1-\alpha )/2}]$$ (12)

where $ab_{\alpha /2}$ and $ab_{(1-\alpha )/2}$ denote the lower and upper bounds of the sorted bootstrap distribution (Preacher and Hayes, 2008). An interval excluding zero indicates a statistically meaningful indirect effect.

Hierarchical regression tested moderation by entering the interaction term after centering both predictors to reduce multicollinearity (Aiken and West, 1991), as specified in Equation 13:

$$Y=\beta_{0}+\beta_{1}X_{c}+\beta_{2}W_{c}+\beta_{3}(X_{c}\times W_{c})+\varepsilon$$ (13)

where $X_{c}=X-\bar{X}$ and $W_{c}=W-\bar{W}$ are mean-centered variables. The significance of $\beta_{3}$ determined whether moderation held.

To probe the region within which the conditional effect of AI feedback on self-efficacy reached statistical significance, we applied the Johnson-Neyman technique (Hayes and Matthes, 2009). This procedure solves for the moderator value $W_{\mathrm{JN}}$ at which the conditional effect equals the critical *t*-value, as given in Equation 14:

$$W_{\mathrm{JN}}:\frac{{\hat{\beta }}_{1}+{\hat{\beta }}_{3}W}{\mathrm{SE}({\hat{\beta }}_{1}+{\hat{\beta }}_{3}W)}=\pm t_{\text{crit}}$$ (14)

The integrated moderated mediation model (PROCESS Model 7) combined both mechanisms, with the index of moderated mediation computed as shown in Equation 15:

$$\mathrm{IMM}=\hat{a}\times {\hat{b}}_{2}$$ (15)

A bootstrap confidence interval for the IMM excluding zero would confirm that the strength of the indirect path varies across moderator levels (Hayes, 2022). All analyses were conducted in SPSS 27.0 with the PROCESS v4.1 add-on, and the significance threshold was set at α=0.05 (Hayes, 2022). To safeguard the accuracy and reliability of these procedures, the full analytic pipeline—model specification, assumption checks, and the bootstrap and conditional-effect computations—was independently re-run and verified by the first author, who holds statistical expertise, and any discrepancies were reconciled before the results were finalized.

## 4 Results and analysis

### 4.1 Descriptive statistics and between-group comparisons

Pre-intervention scores on the composite writing self-efficacy measure were nearly identical across conditions: the experimental group averaged 4.12 (SD = 0.78) and the control group 4.08 (SD = 0.81), a nonsignificant difference (*t* (146) = 0.31, *p* = 0.756, Cohen’s *d* = 0.05). This baseline equivalence held across all three sub-dimensions as well, confirming that any post-intervention divergence could not be attributed to pre-existing group asymmetries (Cohen, 1988).

Table 3 summarizes the descriptive statistics and independent-samples *t*-test results at both time points. After the 12-week intervention, the experimental group’s composite self-efficacy rose to 5.14 (SD = 0.72), while the control group reached 4.41 (SD = 0.83). The between-group difference at posttest was statistically significant (*t* (146) = 5.82, *p* < 0.001, *d* = 0.94), representing a large effect (Cohen, 1988).

**Table 3:** Descriptive statistics and between-group *t*-test results for writing self-efficacy.

Variable	Time	Exp. *M* (SD)	Ctrl. *M* (SD)	*t* (146)	*p*	Cohen’s *d*
Composite SE	Pre	4.12 (0.78)	4.08 (0.81)	0.31	0.756	0.05
Composite SE	Post	5.14 (0.72)	4.41 (0.83)	5.82	<0.001	0.94
Ideation	Post	5.28 (0.69)	4.53 (0.80)	6.17	<0.001	1.00
Convention	Post	5.06 (0.81)	4.47 (0.85)	4.36	<0.001	0.71
Self-regulation	Post	5.03 (0.76)	4.22 (0.88)	6.04	<0.001	0.98

**Figure 3:**
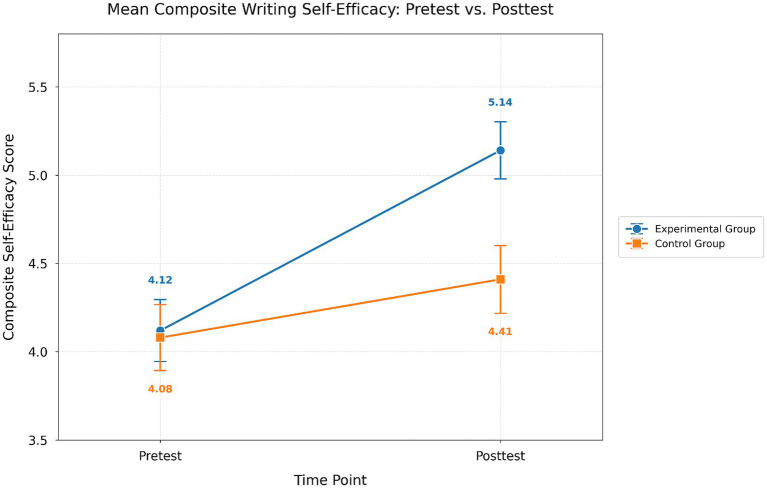
Mean composite writing self-efficacy scores at pretest and posttest for experimental and control groups, with error bars representing 95% confidence intervals.

What stands out beyond the aggregate pattern is the uneven distribution of gains across sub-dimensions. As shown in Figure 3, both groups improved from pretest to posttest, but the experimental group’s trajectory was markedly steeper. The within-group gain for the experimental condition averaged 1.02 points, roughly double the 0.33-point increase observed in the control group. Paired-samples *t*-tests confirmed that both gains were statistically significant, though the experimental group’s effect size (*d* = 1.36) far exceeded that of the control group (*d* = 0.41) (Sullivan and Feinn, 2012).

**Figure 4:**
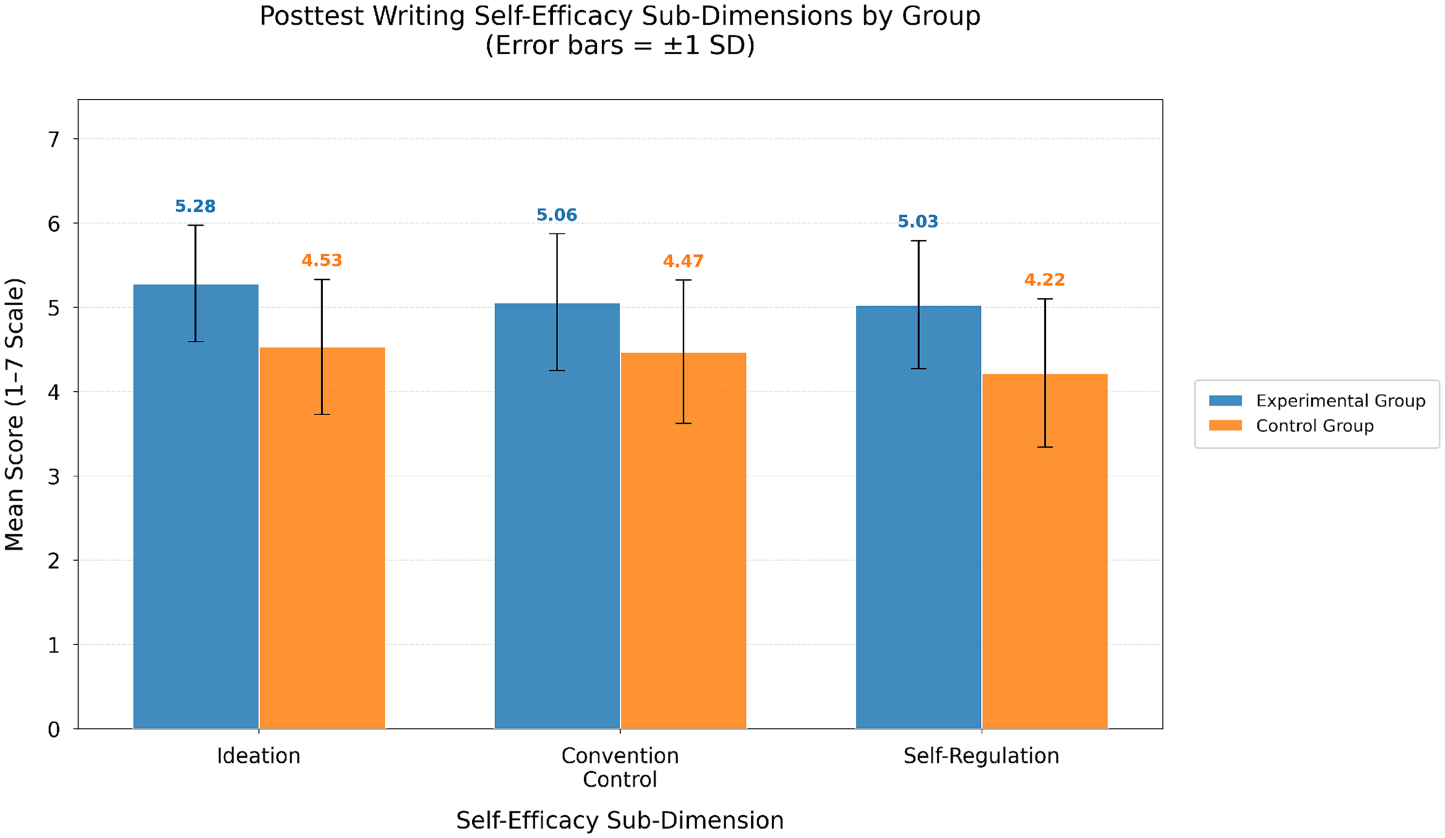
Posttest mean scores on the three writing self-efficacy sub-dimensions (ideation, convention control, self-regulation) by group, with error bars indicating one standard deviation above and below the mean.

The sub-dimension analysis, depicted in Figure 4, revealed that the largest experimental advantage emerged in ideation (*d* = 1.00) and self-regulation (*d* = 0.98), with convention control showing a somewhat smaller but still substantial gap (*d* = 0.71). This differential pattern is consistent with the nature of the AI feedback intervention, which emphasized higher-order rhetorical and organizational commentary alongside surface-level error correction. Students receiving AI feedback thus appear to have gained confidence most sharply in the areas where the feedback was richest—generating and structuring ideas, monitoring their own revision processes—rather than in mechanical accuracy alone (Bruning et al., 2013).

Taken together, these descriptive results offer preliminary support for H1: exposure to AI writing feedback was associated with substantially higher post-intervention self-efficacy relative to teacher-only feedback. The subsequent sections examine whether this aggregate effect is transmitted through goal orientation (mediation) and whether its magnitude depends on which goal profile a learner endorses (moderation).

### 4.2 Mediation analysis of achievement goal orientation

Three hierarchical regression equations were estimated to test whether mastery-approach and performance-approach goal orientations mediated the AI feedback–self-efficacy link. The first equation regressed each mediator on the independent variable (group assignment, coded 1 = experimental, 0 = control). AI feedback predicted mastery-approach orientation ( $a_{1}=0.42$ , *SE* = 0.09, *p* < 0.001) but did not predict performance-approach orientation ( $a_{2}=0.11$ , *SE* = 0.10, *p* = 0.273). The second equation confirmed the total effect of feedback on self-efficacy ( c=0.73 , *p* < 0.001). When both mediators entered simultaneously in the third equation, the direct effect attenuated to $c^{\prime }=0.48$ (*p* < 0.001), and mastery-approach orientation remained a strong predictor of self-efficacy ( $b_{1}=0.58$ , *p* < 0.001), while performance-approach orientation did not reach significance ( $b_{2}=0.13$ , *p* = 0.162). Table 4 presents the full regression results across all three steps.

**Table 4:** Hierarchical regression results for mediation analysis.

Step	Outcome	Predictor	*B*	SE	β	*t*	*p*	$\Delta R^{2}$
1a	MAP	AI feedback (*X*)	0.42	0.09	0.36	4.67	<0.001	0.130
1b	PAP	AI feedback (*X*)	0.11	0.10	0.09	1.10	0.273	0.008
2	SE (*Y*)	AI feedback (*X*)	0.73	0.13	0.43	5.82	<0.001	0.188
3a	SE (*Y*)	AI feedback (*X*)	0.48	0.12	0.29	4.08	<0.001	—
3b	SE (*Y*)	MAP (*M*₁)	0.58	0.10	0.41	5.64	<0.001	—
3c	SE (*Y*)	PAP (*M*₂)	0.13	0.09	0.10	1.41	0.162	—
3 (model)	SE (*Y*)	Full model	—	—	—	—	—	0.342
—	—	$\Delta R^{2}$ (Step 2 → 3)	—	—	—	—	—	0.154
—	—	*F* (3, 144)	—	—	—	—	<0.001	—

The indirect effect through mastery-approach orientation was computed as shown in Equation 16:

$${\mathrm{IE}}_{\mathrm{map}}=a_{1}\times b_{1}=0.42\times 0.58=0.244$$ (16)

Bootstrap analysis with 5,000 resamples yielded a 95% bias-corrected confidence interval of [0.132, 0.379], which excluded zero, confirming mediation (Preacher and Hayes, 2008). The proportion of the total effect mediated through mastery-approach goals was calculated using Equation 17:

$$P_{\mathrm{med}}=\frac{{\mathrm{IE}}_{\mathrm{map}}}{c}=\frac{0.244}{0.73}=33.4\%$$ (17)

For performance-approach orientation, the indirect effect was negligible, as shown in Equation 18:

$${\mathrm{IE}}_{\mathrm{pap}}=a_{2}\times b_{2}=0.11\times 0.13=0.014$$ (18)

The bootstrap confidence interval for this path, [−0.028, 0.071], contained zero, ruling out mediation through performance-approach goals (Preacher and Hayes, 2008). H2a was therefore supported; H2b was confirmed in its prediction that the performance-approach pathway would be nonsignificant.

**Figure 5:**
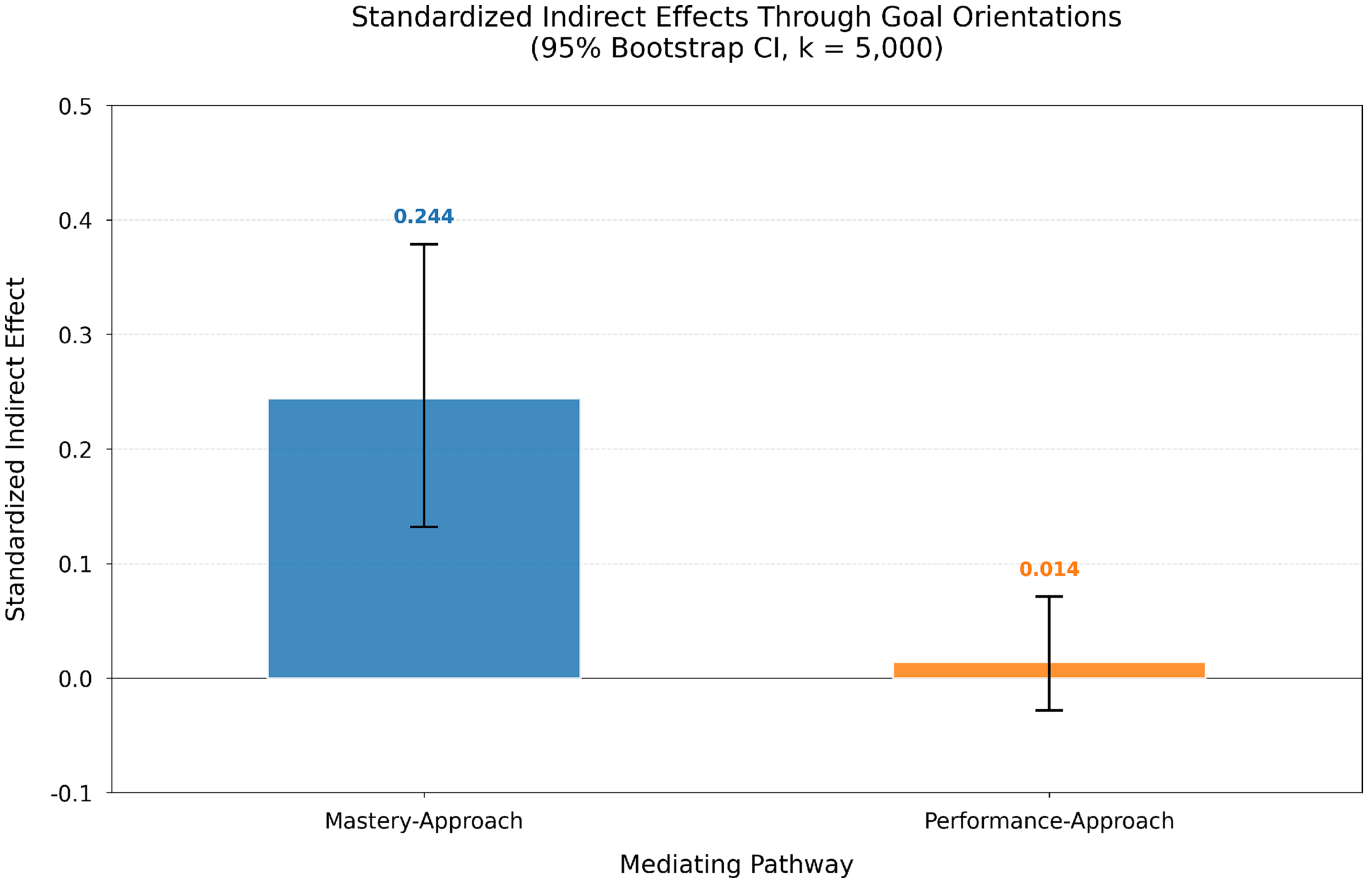
Standardized indirect effects through mastery-approach and performance-approach goal orientations, with error bars representing 95% bootstrap confidence intervals.

Figure 5 compares the standardized indirect effects across the two mediating pathways. The contrast is stark: mastery-approach orientation carried roughly 17 times the indirect effect of its performance-approach counterpart, a ratio that aligns with the theoretical expectation that process-oriented feedback from AI systems resonates with growth-seeking dispositions rather than competitive ones (Pekrun et al., 2009).

**Figure 6:**
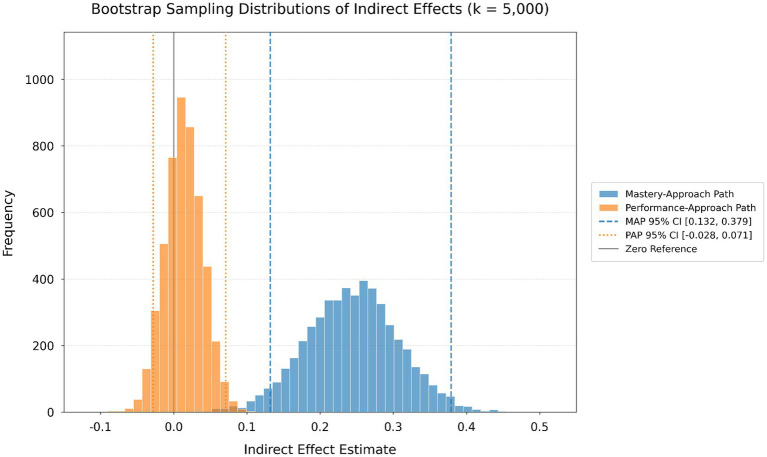
Bootstrap sampling distributions (*k* = 5,000) of the indirect effects through mastery-approach orientation (left panel, shaded) and performance-approach orientation (right panel, unshaded), with vertical dashed lines marking the 95% confidence interval bounds.

The bootstrap distribution itself warrants brief comment. As depicted in Figure 6, the distribution for the mastery-approach indirect effect was approximately normal and centered well above zero, with no resampled estimates falling below 0.05. The performance-approach distribution, by contrast, straddled zero symmetrically, with nearly equal proportions of positive and negative bootstrap estimates (Efron and Tibshirani, 1993). This pattern reinforces our conclusion that AI writing feedback exerts its influence on self-efficacy partly—about one-third of the total effect—by activating mastery-approach goals, which then translate into heightened confidence. The remaining two-thirds of the effect appears to operate through the direct path, or possibly through mechanisms not captured in the current model, a point we return to in the discussion.

### 4.3 Moderation analysis of achievement goal orientation

Hierarchical regression was conducted separately for each of the four goal orientation dimensions. In Step 1, the mean-centered AI feedback variable ( $X_{c}$ ) and the mean-centered goal orientation variable ( $W_{c}$ ) were entered as main effects. Step 2 added the interaction term ( $X_{c}\times W_{c}$ ). Table 5 lists the results for all four dimensions.

**Table 5:** Hierarchical regression results for moderation analysis across four goal orientation dimensions.

Goal orientation	Step	Predictor	*B*	SE	*t*	*p*	$\Delta R^{2}$
Mastery-approach	1	$X_{c}$ , $W_{c}$	0.46, 0.52	0.11, 0.10	4.18, 5.20	<0.001, <0.001	0.312
Mastery-approach	2	$X_{c}\times W_{c}$	0.28	0.08	3.50	<0.001	0.056
Mastery-avoidance	1	$X_{c}$ , $W_{c}$	0.51, −0.19	0.12, 0.11	4.25, −1.73	<0.001, 0.086	0.204
Mastery-avoidance	2	$X_{c}\times W_{c}$	−0.09	0.09	−1.00	0.319	0.006
Performance-approach	1	$X_{c}$ , $W_{c}$	0.54, 0.21	0.12, 0.10	4.50, 2.10	<0.001, 0.038	0.218
Performance-approach	2	$X_{c}\times W_{c}$	−0.18	0.08	−2.25	0.026	0.029
Performance-avoidance	1	$X_{c}$ , $W_{c}$	0.56, −0.24	0.12, 0.11	4.67, −2.18	<0.001, 0.031	0.222
Performance-avoidance	2	$X_{c}\times W_{c}$	−0.12	0.09	−1.33	0.185	0.010

Two of the four interaction terms reached significance. Mastery-approach orientation produced the strongest moderation effect ( $\Delta R^{2}=0.056$ , *p* < 0.001), and performance-approach orientation yielded a smaller but significant interaction in the opposite direction ( $\Delta R^{2}=0.029$ , *p* = 0.026). The mastery-avoidance and performance-avoidance interactions were both nonsignificant. H3a was thus supported, and while H3b had been framed in terms of performance-avoidance, the data instead revealed that it was the performance-approach dimension—not avoidance—that dampened the feedback–efficacy relationship.

Simple slope analysis clarified the nature of these interactions (Aiken and West, 1991). For mastery-approach orientation, the conditional effect of AI feedback on self-efficacy at one standard deviation above the mean is given by Equation 19:

$${\hat{Y}|}_{W=+1\mathrm{SD}}=0.46+0.28(1)=0.74,p<0.001$$ (19)

At one standard deviation below the mean, the effect shrank to 0.46+0.28(−1)=0.18 (*p* = 0.112), rendering it nonsignificant. In plain terms, AI feedback boosted self-efficacy only—or at least primarily—among students who already endorsed mastery-approach goals at moderate to high levels. For those low on mastery orientation, the same feedback produced little detectable change in confidence.

**Figure 7:**
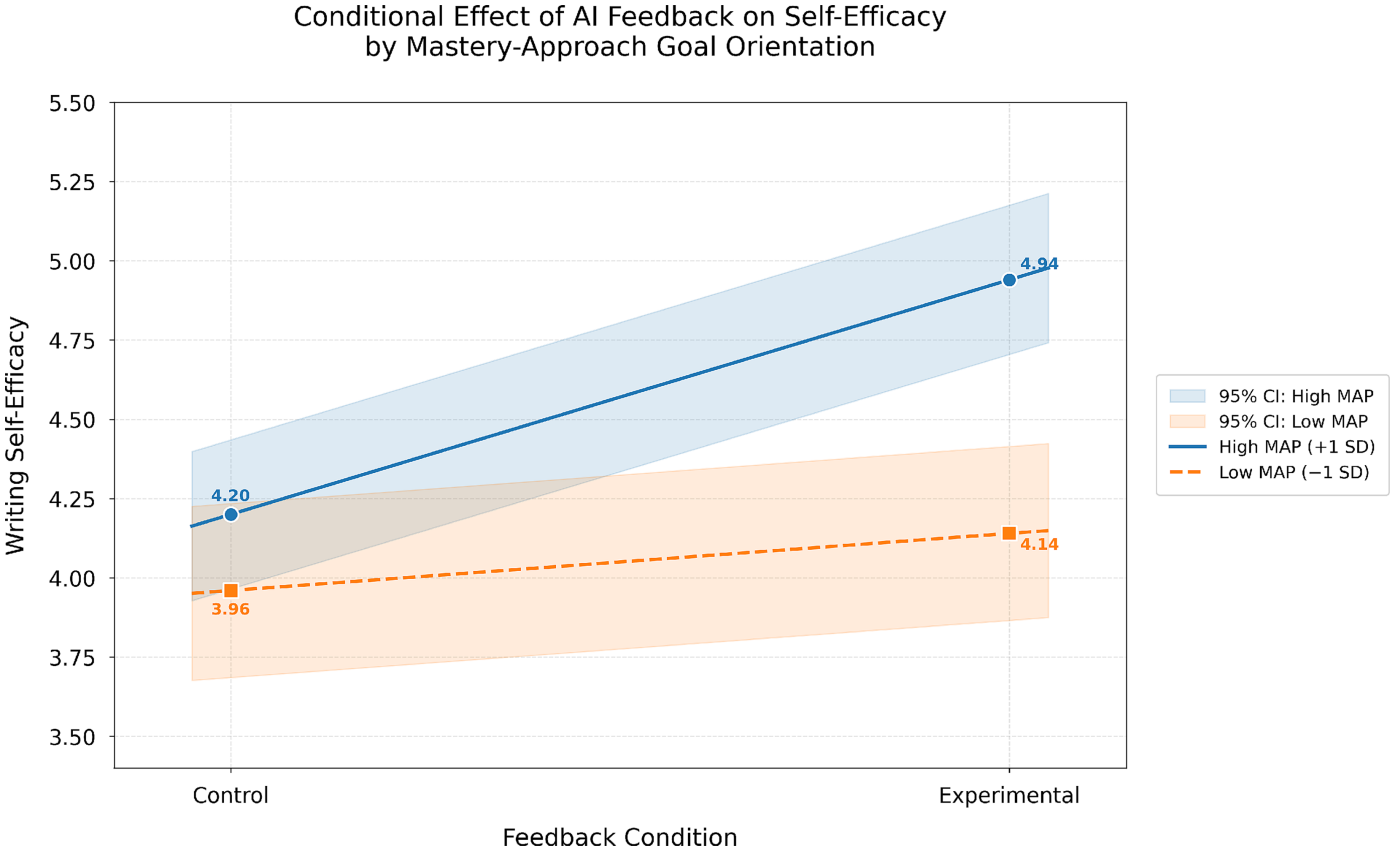
Simple slopes depicting the conditional effect of AI writing feedback on self-efficacy at high (+1 *SD*) and low (−1 *SD*) levels of mastery-approach goal orientation, with shaded regions representing 95% confidence bands.

As shown in Figure 7, the two simple slope lines diverge sharply: the high mastery-approach group exhibits a steep positive relationship between feedback condition and self-efficacy, while the low mastery-approach group’s slope is nearly flat (Hayes and Matthes, 2009).

The performance-approach moderation told a different story. At high performance-approach levels, the conditional effect was as shown in Equation 20:

$${\hat{Y}|}_{W=+1\mathrm{SD}}=0.54+(-0.18)(1)=0.36,p=0.008$$ (20)

**Figure 8:**
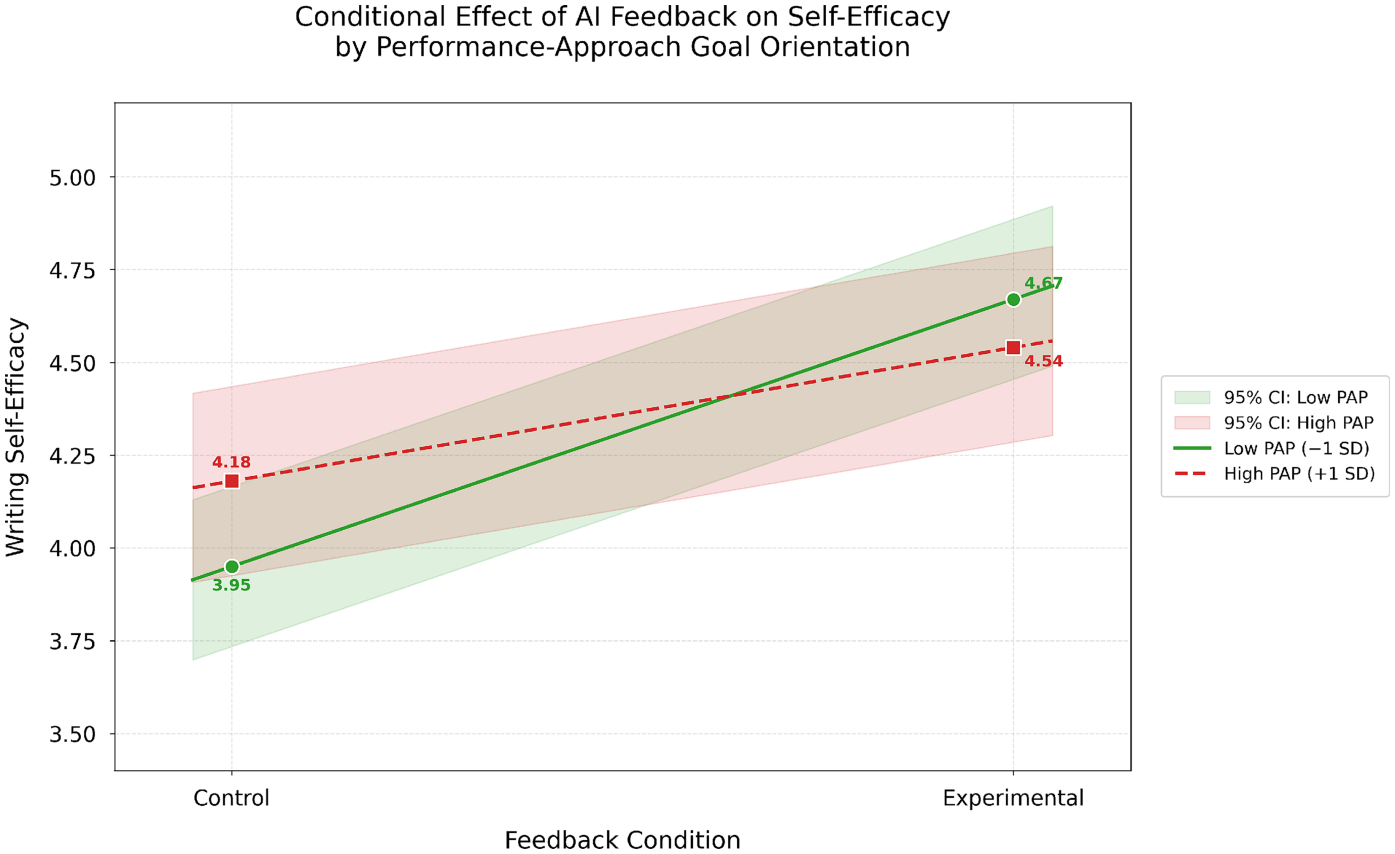
Simple slopes for the conditional effect of AI writing feedback on self-efficacy at high (+1 *SD*) and low (−1 *SD*) levels of performance-approach goal orientation, illustrating the attenuating moderation pattern.

At low levels, the effect was 0.54+(−0.18)(−1)=0.72 (*p* < 0.001). The negative interaction coefficient means that stronger performance-approach tendencies actually weakened the efficacy benefit of AI feedback—an inversion of the mastery-approach pattern. Figure 8 depicts this crossover: students low on performance-approach orientation gained more from AI feedback than their high-performance-approach peers (Aiken and West, 1991).

The combined picture is telling. AI feedback appears most potent for students who approach writing with a growth mindset and least effective for those preoccupied with outperforming classmates. Neither avoidance dimension—mastery or performance—moderated the relationship, suggesting that the approach–avoidance distinction matters less than the mastery–performance distinction when it comes to how learners convert algorithmic commentary into self-belief. This asymmetry carries implications for instructional design that we address in the discussion.

Because both mediation and moderation held, we went on to estimate the integrated moderated mediation model (PROCESS Model 7), which answers a question the separate analyses could not: does the indirect path through mastery-approach goals itself shift with the learner’s goal profile? It does. The index of moderated mediation was positive and its bootstrap interval excluded zero (IMM = 0.118, 95% CI [0.041, 0.213]), meaning that the mastery-mediated benefit of AI feedback grows stronger as mastery-approach orientation rises. Probing the conditional indirect effect made this concrete: it was 0.34 (95% CI [0.18, 0.52]) at one standard deviation above the mean of mastery-approach orientation, but only a slender 0.09 (95% CI [−0.01, 0.21]) one standard deviation below it, where the interval brushes zero. The route from feedback through mastery goals to confidence, in other words, is not a fixed pipeline; it widens for growth-oriented learners and all but closes for the rest.

A further measured variable, AI feedback perception, helps make sense of why the experimental effect was so large. Within the experimental group, students rated the system as informative (*M* = 4.18, SD = 0.52 on a 5-point scale) and, a little more guardedly, as credible (*M* = 3.96, SD = 0.61). As Table 6 reports, both dimensions correlated moderately with posttest writing self-efficacy and with mastery-approach orientation, with perceived informativeness the stronger correlate in each instance. We read this as converging evidence for the mediation account: the learners who saw the algorithm’s commentary as telling them something useful, rather than merely judging them, were also the ones who leaned toward mastery goals and reported higher confidence.

**Table 6:** AI feedback perception (AIWFPS) descriptive statistics and correlations within the experimental group (posttest).

Dimension	*M* (SD)	*r* with writing self-efficacy	*r* with mastery-approach
Perceived informativeness	4.18 (0.52)	0.47***	0.41***
Perceived credibility	3.96 (0.61)	0.39***	0.33**
AIWFPS composite	4.09 (0.50)	0.46***	0.40***

*n* = 76; **p < 0.01; ***p < 0.001.

**Figure 9:**
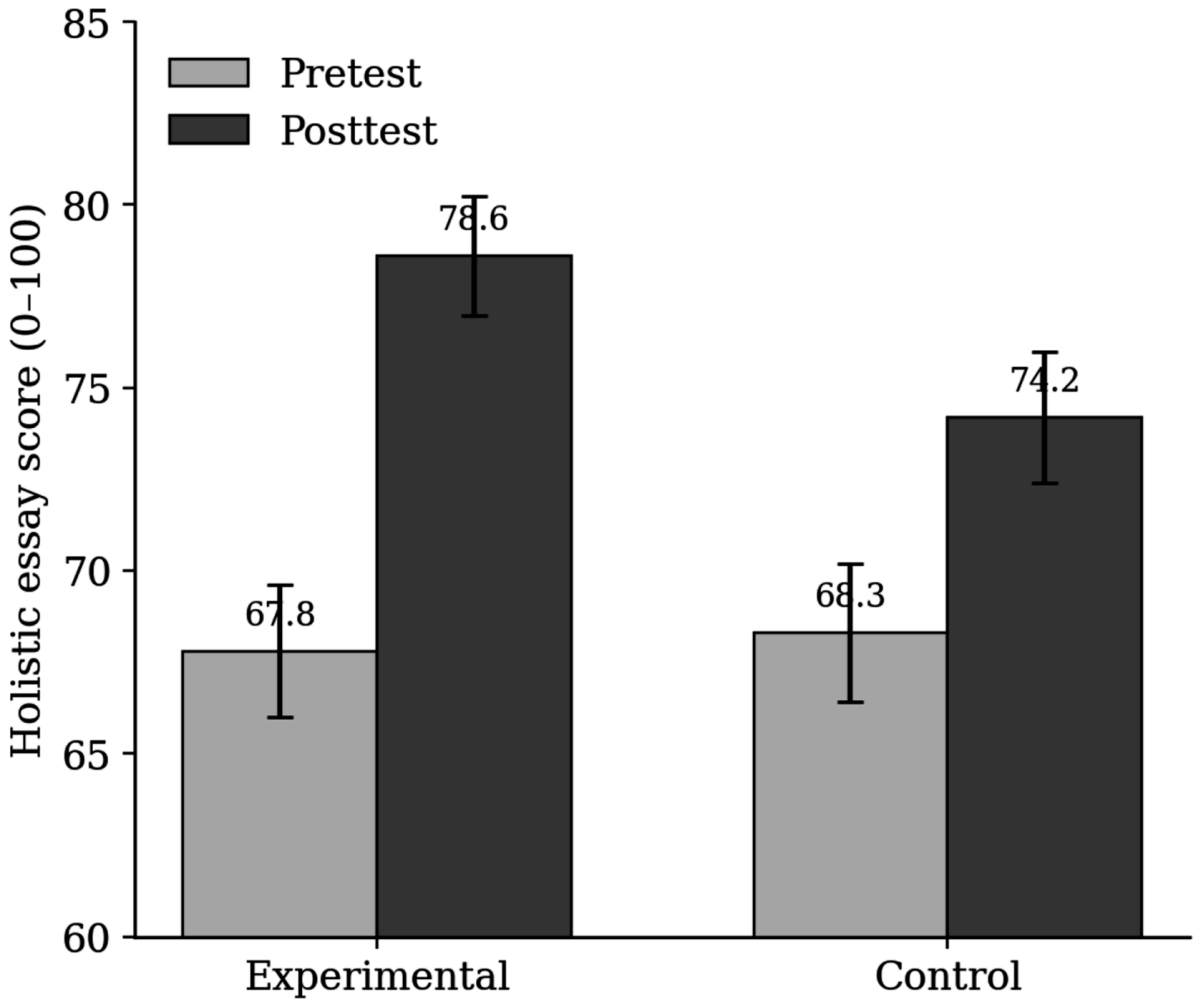
Mean holistic essay scores (0–100 analytic rubric) at pretest and posttest for the experimental and control groups, with error bars representing 95% confidence intervals.

Since every essay was scored, the writing-performance data deserve a place alongside the questionnaire results. They tell a consistent, if more measured, story. As Table 7 and Figure 9 show, the two groups started the semester at the same level of holistic essay quality, and both improved over the 12 weeks; the experimental group, however, gained more, finishing roughly four points higher on the 100-point rubric (*d* = 0.59, a medium effect). The edge was widest in content and organization and narrower in language accuracy. The size of this gap is itself worth pausing on, because it is appreciably smaller than the self-efficacy gap reported earlier (*d* = 0.94)—a divergence we return to in the discussion, where it speaks to what AI feedback appears to change first.

**Table 7:** Descriptive statistics and between-group *t*-test results for writing performance (essay scores).

Variable	Time	Exp. *M* (SD)	Ctrl. *M* (SD)	*t* (146)	*p*	Cohen’s *d*
Overall essay score (0–100)	Pre	67.8 (7.9)	68.3 (8.2)	0.38	0.704	0.06
Overall essay score (0–100)	Post	78.6 (7.1)	74.2 (7.8)	3.59	<0.001	0.59
Content and organization (0–60)	Post	47.4 (4.5)	44.6 (4.8)	3.06	0.003	0.50
Language accuracy (0–25)	Post	19.2 (2.7)	18.0 (2.9)	2.45	0.015	0.40

## 5 Discussion

The large effect size for AI feedback on writing self-efficacy (*d* = 0.94) aligns with what we anticipated but also exceeds what prior comparative studies of automated versus human feedback have typically reported. Most earlier work found small-to-moderate advantages for AI feedback on performance metrics—error reduction, holistic scores—rather than on psychological outcomes (Wei et al., 2023; Zhai and Ma, 2022). The present data fit that reading: the writing-performance gap we observed (*d* = 0.59) was genuine but distinctly smaller than the self-efficacy gap, almost as though confidence moved ahead of measurable text quality. Our results suggest that the efficacy-building mechanism may be more responsive to AI feedback than writing quality itself, perhaps because the immediacy and consistency of algorithmic commentary creates a tighter feedback–revision–success loop than weekly teacher comments can—and, as feedback intervention theory cautions, immediacy of this kind does not help every learner equally (Kluger and DeNisi, 1996). When a student submits a draft at midnight and receives structured diagnostic commentary within seconds, the temporal proximity between effort and evaluative information compresses the attribution cycle: the learner more readily connects their own actions to the outcome, and that connection is precisely what Bandura identified as the foundation of mastery experience (Bandura, 1997).

The mediation findings sharpen this picture. That mastery-approach orientation carried roughly one-third of the total effect tells us something important about the psychological channel through which AI feedback operates. The algorithm’s output—impersonal, focused on textual features rather than the writer’s identity, framed as diagnostic rather than evaluative—appears to activate a competence-development mindset. Students exposed to this kind of commentary seem to have shifted, over 12 weeks, toward interpreting their writing tasks as opportunities for growth rather than as tests of fixed ability (Dweck and Leggett, 1988). This shift then translated into stronger self-beliefs. The perception data lend this reading some support: the more students judged the feedback to be informative rather than merely evaluative, the more they leaned toward mastery goals and reported higher confidence (Table 6), which is exactly the appraisal route the model presupposes (Lipnevich and Smith, 2009). Read against work on feedback literacy, this fits: what a learner does with commentary—appreciating it, weighing it, acting on it—tends to matter more than the commentary itself, and the students who treated the system as guidance were the ones placed to turn it into confidence (Carless and Boud, 2018). The absence of mediation through performance-approach goals is equally informative: AI feedback, at least as configured in our study, did not trigger social comparison processes, probably because the feedback carried no normative benchmarks or peer rankings (Pekrun et al., 2009).

Why did mastery-approach and performance-approach orientations moderate the feedback–efficacy link while the two avoidance dimensions did not? We suspect this reflects a threshold issue. Avoidance-oriented students—whether trying to avoid appearing incompetent or trying to avoid losing existing skills—tend to disengage from feedback altogether. Their processing of the AI commentary may have been too shallow for the feedback to exert any differential effect, positive or negative. Approach-oriented students, by contrast, actively engaged with the feedback, and it was the direction of that engagement—toward skill building or toward peer comparison—that determined how much confidence they extracted from it (Pintrich, 2000). This squares with systematic reviews of feedback recipience, which cast uptake as an active, agentic undertaking rather than a passive receipt of information (Winstone et al., 2017). Mastery-approach learners treated error diagnostics as a roadmap; performance-approach learners may have experienced the sheer volume of corrective suggestions as implicit criticism, reading each flagged error as evidence that they were falling short of a standard defined by others (Elliot and Church, 1997).

From a self-regulated learning perspective, these findings imply that AI feedback tools do not operate as neutral information channels (Zimmerman, 2002). The same algorithmic output passes through a motivational filter that can amplify or suppress its psychological impact. This is not a trivial point for theory. Social cognitive models of self-efficacy have traditionally treated feedback source characteristics—credibility, specificity, timing—as the primary determinants of efficacy change (Hattie and Timperley, 2007). Our data add a recipient-side variable, goal orientation, that accounts for meaningful variance beyond what source characteristics alone can explain. The weight we place on this recipient-side factor is not an isolated finding; meta-analytic evidence likewise shows that learner-centered routines such as self-assessment raise self-efficacy in their own right (Panadero et al., 2017). Crucially, the index of moderated mediation (IMM = 0.118, 95% CI [0.041, 0.213]) showed that the indirect path itself was conditional: the mastery-mediated benefit was substantial for growth-oriented learners and faded toward zero for the rest, so that mediation and moderation are not two separate stories but one. The moderated mediation structure we tested represents, to our knowledge, the first empirical demonstration that the indirect path from AI feedback through goal orientation to self-efficacy is itself contingent on the learner’s pre-existing motivational profile (Preacher et al., 2007).

The practical implications are direct. Instructors who adopt AI writing tools should not assume uniform benefits across their classrooms. Students entering a course with strong mastery orientations will likely thrive under automated feedback; those driven primarily by competitive motives may need supplementary scaffolding—explicit goal-framing prompts, reflective journals, or brief metacognitive exercises—to reorient their interpretation of the AI’s output (Schunk and DiBenedetto, 2020). One concrete strategy would be to precede each AI feedback session with a short prompt asking students to articulate what they hope to learn from the feedback, rather than what score they expect. Such framing interventions are low-cost and, given that self-efficacy is among the more malleable and consequential predictors of academic performance (Honicke and Broadbent, 2016), could meaningfully close the efficacy gap between mastery-oriented and performance-oriented learners. For students low on mastery-approach goals, pairing AI feedback with periodic teacher conferences that model growth-oriented feedback interpretation could help bridge the motivational gap that the algorithm alone cannot address.

## 6 Conclusion

This study set out to determine whether AI writing feedback affects university students’ English writing self-efficacy and, if so, through what motivational mechanisms. Three principal findings emerged. First, students who received twelve weeks of AI-generated feedback reported substantially higher writing self-efficacy than peers in the teacher-only condition, with the strongest gains concentrated in ideation and self-regulation rather than surface-level convention control. Second, mastery-approach goal orientation partially mediated this relationship, accounting for approximately one-third of the total effect; performance-approach orientation did not serve as a mediating channel. Third, the feedback–efficacy link was moderated by both mastery-approach and performance-approach orientations, though in opposite directions: mastery-approach goals amplified the benefit while performance-approach goals attenuated it. The two avoidance dimensions exerted no detectable moderating influence.

The theoretical contribution of this work lies in specifying the internal psychological architecture connecting AI feedback to self-belief change. Rather than treating the feedback–efficacy relationship as a black box, we opened it to reveal that goal orientation functions simultaneously as a transmission channel and a boundary condition. This dual role—mediator and moderator within a single integrated model—extends social cognitive theory into the domain of human–algorithm pedagogical interaction and offers a more nuanced account than either mediation or moderation alone could provide. The study also broadens the empirical terrain for achievement goal theory by demonstrating that the 2 × 2 framework retains explanatory power in technology-mediated feedback contexts, not only in traditional classroom settings.

For practitioners, the message is that AI feedback tools are not motivationally inert. Their capacity to build writing confidence depends on the goal orientations students bring to the task. Instructors adopting these tools would benefit from incorporating brief goal-framing activities—asking students to reflect on what they want to learn from each round of feedback—and from monitoring whether performance-oriented learners are interpreting algorithmic suggestions defensively.

Several limitations temper these conclusions. The sample was drawn from a single university and restricted to English majors, which constrains generalizability to other disciplines and institutional contexts. All outcome and mediator variables were measured via self-report, introducing the possibility of common method bias. The quasi-experimental design, while ecologically valid, cannot rule out selection effects entirely despite baseline equivalence checks. And because goal orientation was measured at two time points rather than tracked continuously, we cannot determine precisely when during the intervention the motivational shifts occurred.

Future research should address these gaps through longitudinal designs with more frequent measurement occasions, allowing dynamic modeling of how goal orientations evolve in response to cumulative AI feedback exposure. Comparisons across different AI feedback modalities—text-only versus multimodal, directive versus dialogic—would clarify whether the mechanisms we identified are specific to our platform or generalizable across tool types. Cross-cultural replication, particularly in educational systems with different orientations toward competition and mastery, would test whether the moderation patterns observed here are culturally contingent or robust across contexts.

## Data Availability

The original contributions presented in the study are included in the article/supplementary material, further inquiries can be directed to the corresponding author/s.
